# Estimating uncertainty in the volume and carbon storage of downed coarse woody debris

**DOI:** 10.1002/eap.1844

**Published:** 2019-01-28

**Authors:** John L. Campbell, Mark B. Green, Ruth D. Yanai, Christopher W. Woodall, Shawn Fraver, Mark E. Harmon, Mark A. Hatfield, Charles J. Barnett, Craig R. See, Grant M. Domke

**Affiliations:** ^1^ Northern Research Station USDA Forest Service Durham New Hampshire 03824 USA; ^2^ Center for the Environment Plymouth State University Plymouth New Hampshire 03264 USA; ^3^ Department of Forest and Natural Resources Management SUNY College of Environmental Science and Forestry Syracuse New York 13210 USA; ^4^ School of Forest Resources University of Maine Orono Maine 04469 USA; ^5^ Department of Forest Ecosystems and Society Oregon State University Corvallis Oregon 97331 USA; ^6^ Northern Research Station USDA Forest Service Newtown Square Pennsylvania 19073 USA; ^7^ Department of Ecology, Evolution and Behavior University of Minnesota St. Paul Minnesota 55108 USA; ^8^ Northern Research Station USDA Forest Service St. Paul Minnesota 55108 USA

**Keywords:** carbon, coarse woody debris, dead wood, error analysis, forest inventory, Monte Carlo, uncertainty

## Abstract

Downed coarse woody debris, also known as coarse woody detritus or downed dead wood, is challenging to estimate for many reasons, including irregular shapes, multiple stages of decay, and the difficulty of identifying species. In addition, some properties are commonly not measured, such as wood density and carbon concentration. As a result, there have been few previous evaluations of uncertainty in estimates of downed coarse woody debris, which are necessary for analysis and interpretation of the data. To address this shortcoming, we quantified uncertainties in estimates of downed coarse woody debris volume and carbon storage using data collected from permanent forest inventory plots in the northeastern United States by the Forest Inventory and Analysis program of the USDA Forest Service. Quality assurance data collected from blind remeasurement audits were used to quantify error in diameter measurements, hollowness of logs, species identification, and decay class determination. Uncertainty estimates for density, collapse ratio, and carbon concentration were taken from the literature. Estimates of individual sources of uncertainty were combined using Monte Carlo methods. Volume estimates were more reliable than carbon storage, with an average 95% confidence interval of 15.9 m^3^/ha across the 79 plots evaluated, which was less than the mean of 31.2 m^3^/ha. Estimates of carbon storage (and mass) were more uncertain, due to poorly constrained estimates of the density of wood. For carbon storage, the average 95% confidence interval was 11.1 Mg C/ha, which was larger than the mean of 4.6 Mg C/ha. Accounting for the collapse of dead wood as it decomposes would improve estimates of both volume and carbon storage. On the other hand, our analyses suggest that consideration of the hollowness of downed coarse woody debris pieces could be eliminated in this region, with little effect. This study demonstrates how uncertainty analysis can be used to quantify confidence in estimates and to help identify where best to allocate resources to improve monitoring designs.

## Introduction

The remains of dead trees and large branches on the forest floor, known as downed coarse woody debris (DCWD), contribute to the structure and function of forest ecosystems. In recent decades, there has been growing recognition of the influence of DCWD on many ecological characteristics and processes including wildlife habitat (Hagan and Grove 1999, McComb and Lindenmayer 1999, Ucitel et al. 2003), fuel loads and fire behavior (Schoennagel et al. 2004), runoff and erosion (Gurnell et al. 1995), water holding capacity and soil moisture (Harmon and Sexton 1995, Goldin and Hutchinson 2014), seedling establishment and regeneration (Harmon and Franklin 1989), carbon storage (Magnússon et al. 2016), nutrient cycling (Shortle et al. 2012, Yuan et al. 2017), and biodiversity (Freedman et al. 1996, Siitonen 2001, Stokland et al. 2012). Because DCWD decomposes relatively slowly, it represents a long‐term carbon storage pool. Dead wood comprises 8% of the carbon stock in the world's forests (Pan et al. 2011), but quantities vary considerably across and within forest types (Harmon and Hua 1991). In temperate forests of the northeastern United States, DCWD amounts to ~20% of the aboveground biomass (Currie and Nadelhoffer 2002, Fahey et al. 2005, Bradford et al. 2009).

Large accumulations of DCWD are commonly the result of disturbances such as wind, snow and ice, and disease (Sturtevant et al. 1997, Pedlar et al. 2002, Cobb et al. 2012). Because the frequency and severity of these disturbances are expected to increase in the future as a result of global change (Dale et al. 2001), rates of tree mortality and ultimately stocks of DCWD will likely also increase. In addition, DCWD quantity is related to forest successional status, as influenced by aging and competition as well as forest management activities (Spies et al. 1988, Sturtevant et al. 1997, Siitonen et al. 2000, Fraver et al. 2002). Slash from logging operations is a form of DCWD with potential ecological benefits, generating interest in its management, in terms of how much is retained and how it is distributed across the landscape (McCarthy and Bailey 1994, Fraver et al. 2002). Growing demand for woody biomass fuel has heightened concerns that whole‐tree harvesting will reduce DCWD, with consequences for forest regeneration and future production (Janowiak and Webster 2010, Riffell et al. 2011).

Despite its important ecological role and implications for forest management, DCWD has often been overlooked, in part because of the lack of commercial value and the expenses and challenges involved in measuring it. However, monitoring of DCWD is increasing in forest inventory programs that require accurate appraisal of all forest carbon stocks (Woodall et al. 2008, Zhu et al. 2017). For example, as a signatory to the United Nations Framework Convention on Climate Change, the United States provides an annual carbon estimate for the forest sector that includes DCWD (Brown 2002, Woodall et al. 2015). The growing interest in DCWD has led to improved efforts to quantify it (e.g., Woodall et al. 2008, Fraver et al. 2013). However, results from inventories are often reported without the accompanying estimates of uncertainty that are crucial for data analysis and interpretation. Estimates of uncertainty can also be used to improve the efficiency of inventory programs by identifying where best to focus efforts in field surveys.

**Figure 1:**
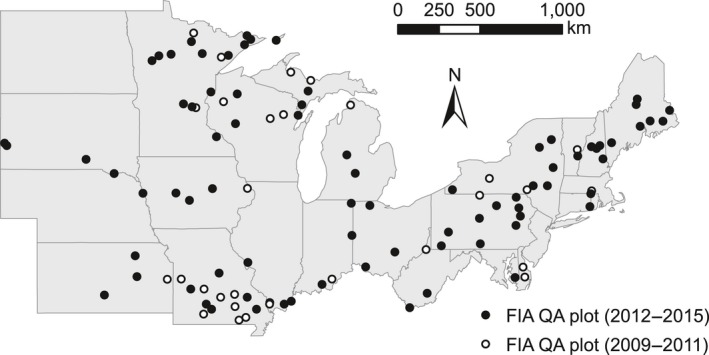
Forest Inventory and Analysis (FIA) plots in which quality assurance has been performed (QA) in the northeastern United States. Open circles are the 31 plots sampled from 2009–2011 using the initial protocol and solid circles are the 79 plots sampled from 2012–2015 using the most recent protocol.

The Forest Inventory and Analysis (FIA) program of the USDA Forest Service conducts and maintains a comprehensive inventory of the forests of the United States. The Northern Research Station FIA unit is responsible for inventorying forests in 24 states in the northeastern United States (Fig. 1). In this region, forests cover more than 70 million ha or about 30% of the land (Oswalt et al. 2014). Although the FIA program dates back to the 1920s, DCWD inventories have been performed only since 2002.

As part of the inventory, the FIA program implements quality assurance (QA) procedures for the data it produces. Around the time the standard “production” crews inventory plots, highly trained and experienced “quality assurance” (QA) crews, composed of field crew supervisors and trainers, make independent inventories (i.e., blind remeasurement) on a subset (2–5% of plots where DCWD is measured) of the same plots using identical methods. Data from the QA crews are used to assess the adequacy of production crew work, check on the performance of individuals, identify areas of improvement where additional training may be warranted, and ensure that the overall forest inventory data collected by FIA are of the desired quality.

We capitalized on this comprehensive and unique QA data set to estimate uncertainty in the volume and carbon storage of DCWD across a suite of FIA plots in the northeastern United States (Fig. 1). We quantified uncertainties in the FIA field measurements and evaluated other possible sources of uncertainty associated with DCWD calculations, specifically those involving estimation of the density and carbon concentration of decomposing wood and possible changes in diameter as downed dead wood collapses. We then produced an overall estimate of uncertainty using a Monte Carlo approach that combined estimates of individual sources of uncertainty for DCWD field measurements and calculations. Finally, we determined how much each individual source of uncertainty contributed to the total DCWD uncertainty estimate. This analysis shows how the inclusion of estimates of uncertainty in DCWD inventories can quantify confidence in the values. It also demonstrates how estimates of uncertainty can be used to identify which aspects of DCWD inventories most need improvement and where best to allocate resources to reduce uncertainty.

## Methods

The USDA Forest Service FIA program conducts its forest inventory on permanent field plots distributed across the United States in a hexagonal grid with a spatial sampling intensity of ~1 plot per 24 km^2^ (6,000 acres; Brand et al. 2000). Downed coarse woody debris is measured as part of a broader suite of forest health attributes that have been monitored since 2002 on a subset of these plots (Woodall and Monleon 2008, USDA Forest Service 2017). In 2012, there was a change in DCWD sampling protocols that reduced the sampling intensity within each plot (reducing DCWD transect length and eliminating measurement of whole logs) in favor of increased spatial sampling intensity (from ~1 plot per 389 km^2^ [96,000 acres] prior to 2012 to ~1 plot per 194 km^2^ (48,000 acres) after 2012). More plots were needed for scaling plot‐level DCWD data to broader spatial extents (e.g., forest type, region, state; Domke et al. 2013, Woodall et al. 2013).

In this analysis, we used forest inventory data collected by the FIA production and QA crews from 110 plots in the northeastern region of the United States from 2009 through 2015 (Fig. 1). Plot‐scale calculations of uncertainty were conducted only for plots sampled using the most recent protocol (i.e., 2012–2015), which included 79 plots in 20 states. Data prior to 2012 were used to augment the data (for diameter, species, and decay class) used for Monte Carlo sampling. The total number of measurements that could be compared between production and QA crews from 2009–2015 was 467 for diameter and decay class, 458 for species identification and 259 for hollowness. Measurements of hollowness were initiated in 2012 with the change in protocol; therefore, there were fewer comparisons available for analysis.

**Figure 2:**
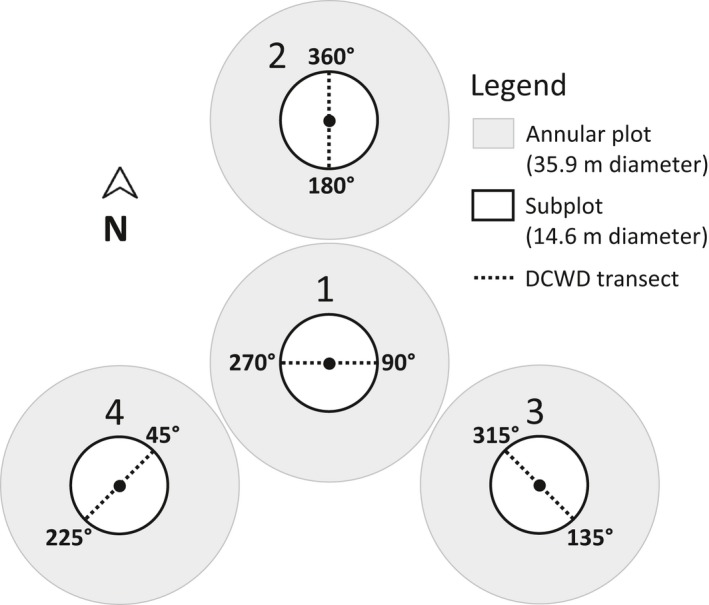
Downed coarse woody debris (DCWD) sampling design for Forest Inventory and Analysis plots that was adopted in 2012. The centers of subplots 2, 3, and 4 are 37 m (120 feet) from the center of subplot 1 at 0°, 120°, and 240° angles, respectively.

Each FIA plot consists of four circular subplots with 14.6 m (48 foot) diameters. Three of the subplots are arranged around a central subplot at a distance of 37 m (120 feet) between plot centers, and at azimuths of 0°, 120°, and 240° (Fig. 2). Downed coarse woody debris is inventoried using the line‐intersect method (Warren and Olsen 1964, Kaiser 1983), which is efficient and highly reproducible (Westfall and Woodall 2007). Under the current sampling protocol, DCWD is inventoried on one 14.6 m long transect centered on each subplot. An individual piece of DCWD is tallied only if its central axis intersects the transect (USDA Forest Service 2017).

The FIA program defines DCWD as boles, large limbs, and other woody pieces that are severed from their original source of growth and are on the ground (Woodall and Monleon 2008, USDA Forest Service 2017). Dead trees (either self‐supported by roots, severed from roots, or uprooted) that are leaning more than 45° from vertical are also included. Our analysis did not include standing dead trees (i.e. less than 45° from vertical), dead shrubs or stumps, downed vegetation that shows signs of life, or downed non‐woody material such foliage or bark that is not an integral part of a bole or limb.

Decay classes follow a five‐class system, with class 1 being least and class 5 most decayed (Table 1; USDA Forest Service 2017). To qualify as DCWD for decay classes 1–4, the piece must be greater than 15 cm (6 inches) in length and greater than 7.6 cm (3 inches) in diameter at the point of intersection. For decay class 5, the piece must have a length of greater than 152 cm (5 feet) exposed at least 13 cm (5 inches) above the forest floor. Smaller mounds of decomposed or buried dead wood are not tallied.

**Table 1:** Description of decay classes for downed coarse woody debris (USDA Forest Service 2017)

Decay class	Structural integrity	Texture of rotten portions	Color of wood	Invading roots	Branches and twigs
1	sound, freshly fallen, intact logs	intact, no rot; stem decay conks absent	original color	absent	if branches are present, fine twigs are still attached and have tight bark
2	sound	mostly intact; sapwood partly soft (starting to decay) but cannot be pulled apart by hand	original color	absent	if branches are present, many fine twigs are gone and remaining fine twigs have peeling bark
3	heartwood sound; piece supports its own weight	hard, large pieces; sapwood can be pulled apart by hand or sapwood absent	reddish‐brown or original color	sapwood only	branch stubs will not pull out
4	heartwood rotten; piece does not support its own weight, but maintains its shape	soft, small blocky pieces; metal pin can be pushed into heartwood	reddish or light brown	throughout	branch stubs pull out
5	none; piece no longer maintains its shape; it is spread out on the ground	soft; powdery when dry	red‐brown to dark brown	throughout	branch stubs and pitch pockets have usually rotted down

For each piece of downed wood that meets these criteria, the following information is collected: diameter at the point of intersection (DI), cavity diameter if hollow (DH), tree species, decay class, and location on the transect. Individual pieces of DCWD are not tagged in FIA inventories; therefore, each piece had to be matched to compare measurements by production and QA crews. To that end, FIA developed a matching algorithm that uses the location along the transect and size of each piece of DCWD to pair the observations (Woodall et al. 2012). The carbon storage of DCWD for each plot is calculated as follows:

(1) $$\begin{array}{cc} & y_{i}=\\ & \frac{\pi^{2}}{8L}\sum_{j=1}^{4}\sum_{m=1}^{2}\sum_{t=1}^{n}\;\left({\text{DI}}_{\mathrm{ijmt}}^{2}-{\text{DH}}_{\mathrm{ijmt}}^{2}\right)\;{\text{CR}}_{\mathrm{ijmt}}\;{\text{BD}}_{\mathrm{ijmt}}\;{\text{DR}}_{\mathrm{ijmt}}\;{\text{CC}}_{\mathrm{ijmt}} \end{array}$$

where $y_{i}=$ downed coarse woody debris carbon storage (Mg C/ha) of plot *i*; L= total transect length of plot *i* (4 × 14.6 m transects = 58.4 m); ${\text{DI}}_{\mathrm{ijmt}}=$ diameter (cm) of piece *t* intersected by transect *m* of subplot *j* of plot *i*; ${\text{DH}}_{\mathrm{ijmt}}=$ diameter of hollow (cm) of piece *t* intersected by transect *m* of subplot *j* of plot *i*; ${\text{CR}}_{\mathrm{ijmt}}=$ collapse ratio of piece *t* intersected by transect *m* of subplot *j* of plot *i*; ${\text{BD}}_{\mathrm{ijmt}}=$ initial bulk density (g/cm^3^) of piece *t* intersected by transect *m* of subplot *j* of plot *i*; ${\text{DR}}_{\mathrm{ijmt}}=$ decay ratio of piece *t* intersected by transect *m* of subplot *j* of plot *i*; and ${\text{CC}}_{\mathrm{ijmt}}=$ carbon concentration of piece *t* intersected by transect *m* of subplot *j* of plot *i*.

The volume of DCWD is calculated with the same formula, but without ${\text{BD}}_{\mathrm{ijmt}}$, ${\text{DR}}_{\mathrm{ijmt}}$ and ${\text{CC}}_{\mathrm{ijmt}}$. A description of the variables and how each source of uncertainty was estimated follows, including the determination of input distributions for the Monte Carlo simulation.

### Field measurements

Diameter measurements (recorded to the nearest inch [2.5 cm] where DCWD crosses the transect) are made using various methods, including calipers, tape measure (circumference when possible or width as determined above the log), and straight‐edge ruler. If it can be ascertained that the piece is hollow where it crosses the transect, the diameter of the cavity is measured to the nearest inch (2.5 cm). If the cavity has an irregular shape or is difficult to measure, a best estimate is recorded. The degree of decay of each DCWD piece is rated according to a five‐class scale (Table 1; Maser et al. 1979, Sollins 1982, USDA Forest Service 2017). When possible, the species of each piece of DCWD is also determined for use in density estimation. However, the species of DCWD can be difficult to identify, especially in advanced stages of decay. In cases where the species cannot be determined, an attempt is made to identify the genus or at least to distinguish hardwoods (angiosperms) from softwoods (gymnosperms). If it is not possible to make that distinction, then the species is recorded as unknown.

### Collapse ratio

**Figure 3:**
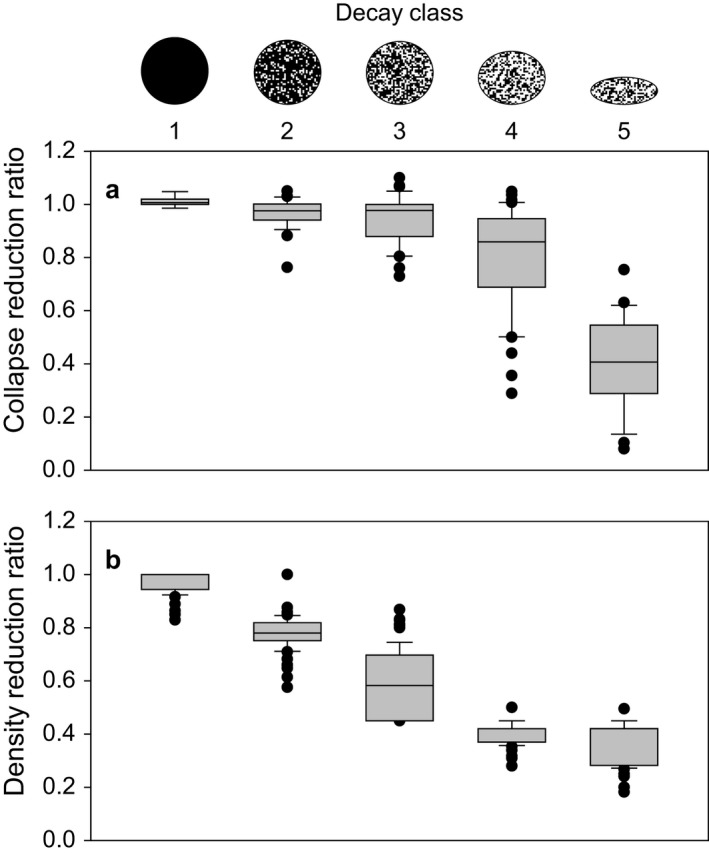
(a) Collapse and (b) density reduction ratios for all species in each decay class showing data from Fraver et al. (2013) for collapse and Harmon et al. (2008) for density. The line within each box is the median; the box represents the interquartile range; the lower and upper error bars are the 10th and 90th percentiles, respectively; and the solid circles are outliers. The ellipses above the graph show the median reduction in height for each decay class, and the fill pattern indicates the median density.

During decomposition, DCWD collapses, and the cross‐section becomes less circular, especially in advanced stages of decay (Fraver et al. 2007). FIA crews typically measure the width (parallel to the forest floor) of decomposed DCWD, not the circumference or vertical height of downed wood pieces. We assessed the importance of DCWD collapse in calculations using collapse ratio data (height : width) from a previous study (Fig. 3a; Fraver et al. 2013). We combined collapse ratio data from the three species studied (*Populus tremuloides* [quaking aspen], *Picea glauc*a [white spruce], and *Pinus resinosa* [red pine]) because data were not available for a broader suite of species, and studies have shown that collapse ratios are fairly consistent across species within decay classes (Fraver et al. 2002, 2013). For each iteration of the Monte Carlo, a collapse ratio value was randomly selected from all the possible values within the decay class (Fraver et al. 2013).

### Density

Estimates of DCWD volume were converted to mass by multiplying by the initial bulk density and a reduction factor (decayed density : undecayed density) that accounts for the decline in density with decay. We used values for both density and density reduction from a synthesis by Harmon et al. (2008) that included wood density data published or collected in North America for 88 tree species, grouped into decay classes (Fig. 3b; Table 1). Of the 54 tree species identified by production and QA crews from 2012–2015, 34% were reported by Harmon et al. (2008), 42% were estimated from other species of the same genera, and 24% were assigned to species based on other criteria because no data were available for the genus (see Data S1). To evaluate uncertainty associated with density reduction, we randomly sampled from the distribution of density reduction factors (defined by the mean and standard error) by species and decay class for each piece of DCWD.

### Biomass to carbon conversion

Carbon storage in DCWD can be determined as the product of the volume, density, and carbon concentration. Harmon et al. (2008) summarized literature values for carbon concentrations of DCWD. Because carbon concentration data for many species are lacking, FIA uses an average value of 49% for hardwoods, 52% for softwoods, and 51% for unknown species types. The standard errors of 0.43% for hardwoods, 0.42% for softwoods, and 0.42% for unknown species were obtained from the Harmon et al. (2008) data by weighting estimates by the number of individuals of each species in each decay class in that data set. For our Monte Carlo simulation, we randomly sampled from these distributions of possible carbon concentration values for hardwood, softwood, and unknown species types.

### Error propagation

The various sources of uncertainty evaluated were combined in a Monte Carlo framework to obtain an overall estimate of uncertainty in DCWD volume and carbon storage. The Monte Carlo analysis involved repeated (10,000 times) random sampling of input variables used in DCWD calculations to estimate the probability distribution of DCWD volume and carbon storage estimates at each plot. Although the Monte Carlo method is computationally intensive, it is relatively straightforward to implement. With this method, the sampled distributions need not be normally distributed, and the error bars may be asymmetrical.

For the diameter and hollowness measurements, which are continuous variables, differences between production and QA crew values were compiled, and an error was randomly sampled from the distributions and added to the actual measurements. For decay class and species identification, which are class variables, each value measured by the production crew was replaced with a randomly selected value from the QA crew corresponding to that class of observations by the field crew.

We used the 95% confidence interval (CI) to represent uncertainty in DCWD, determined as the difference between the 2.5 and 97.5 percentiles of the distribution of possible values. We conducted the Monte Carlo with all the sources of uncertainty combined, and then with only one source of uncertainty at a time to evaluate the relative importance of each individual source of uncertainty. The Monte Carlo analyses were performed in the statistical computing language R (v3.4.1; R Core Team 2017), and the documented computer code and associated source files are available in Data S1. All FIA data, including DCWD, can be downloaded from a publicly accessible database (*available online*).^1^ Additional information about FIA sampling protocols is available at the FIA library (*available online*).^2^

## Results

The mean DCWD volume across plots calculated using the standard FIA method (i.e., Eq. (1) excluding the collapse ratio) was 34.4 m^3^/ha and carbon storage was 4.9 Mg C/ha. When the collapse ratio was included in the DCWD calculation, the mean volume was 9% less (31.2 m^3^/ha) with a range in reduction of 1 to 33% across plots, and the carbon storage was 7% less (4.6 Mg C/ha) with a range of 0 to 30%. The mean DCWD diameter 14 cm and the maximum was 56 cm.

**Figure 4:**
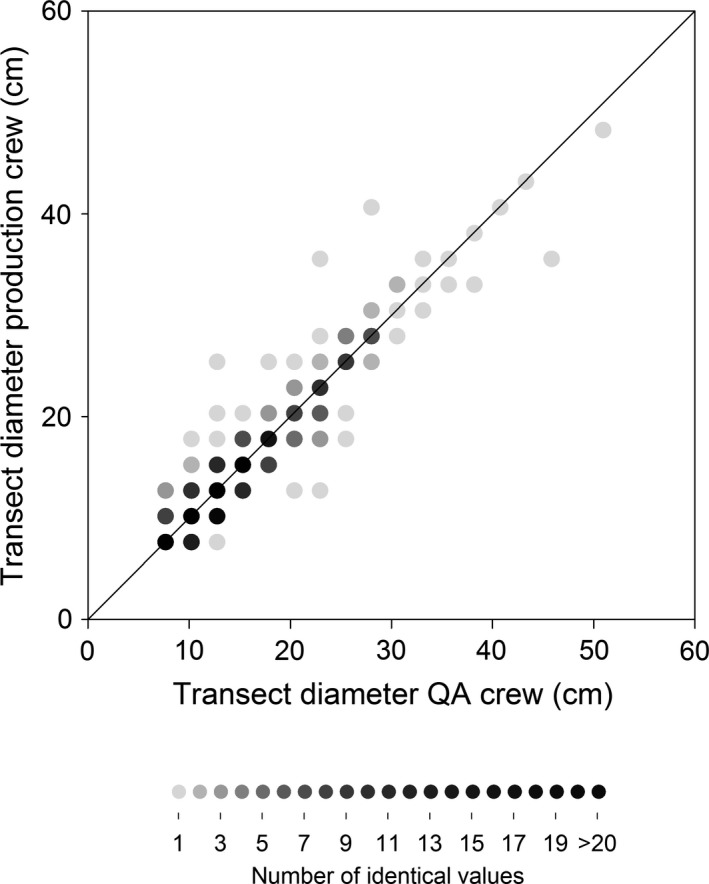
Comparisons of production and QA crew measurements of the diameter of downed coarse woody debris that crosses the transect. The 1:1 line is shown on each plot and the shading indicates the number of identical values. Observations have discrete values because they were recorded to the nearest 2.54 cm (1 inch).

A comparison of diameter measurements between production and QA crews during the entire 2009–2015 period of measurement showed generally close agreement (Fig. 4). For the 467 comparisons, 68% of the diameter measurements matched the precision of the measurement (1 inch), 95% were off by 1 inch (2.5 cm) or less, and 98% by 2 inches (5 cm) or less. The remaining 2% differed by at most 5 inches (12 cm).

**Figure 5:**
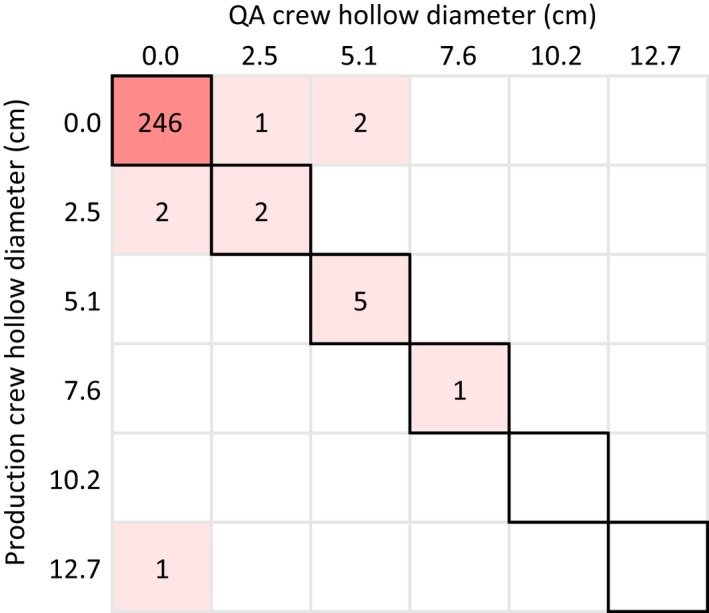
Heat map showing a comparison of the diameter of the hollow part of downed coarse woody debris measured by the QA crew vs. the production crew. Black outlines around cells indicate agreement between the QA and production crews. The value in each cell is the number of pieces of downed coarse woody debris. Measurements are recorded to the nearest 2.54 cm (1 inch).

The production crew identified 5% (25 pieces) of all DCWD as hollow, with a mean cavity diameter of 6 cm and a maximum of 23 cm. For the pieces of DCWD that were hollow, 52% were in decay class 4, 40% were in decay class 3, and 8% were in decay class 2 (Table 2). None of the pieces of DCWD in decay classes 1 or 5 were hollow. Similar to diameter measurements, there was fairly good agreement in measurements of hollowness between production and QA crews (Fig. 5). For trees that were hollow, 57% of cavity diameter measurements matched exactly, 79% were off by 1 inch (2.5 cm) or less, and 93% were off by 2 inches (5 cm) or less. In one case, the difference was more extreme (5 inches, 13 cm).

**Table 2:** For each decay class, the number of downed coarse woody debris pieces, the number of those pieces that were hollow, and the rate of agreement (%) between the QA crew and the production crew in identifying decay class and species

Decay class	Number of pieces		Agreement (%)	
	Total	Hollow	Decay class	Species
1	35	0	89	95
2	86	2	52	89
3	268	10	77	73
4	120	13	65	74
5	11	0	57	—

—, None of the pieces in decay class 5 were identified to the species level by both the production and QA crews.

We evaluated the importance of measuring the hollowness of DCWD by comparing DCWD volume and carbon storage with and without cavity diameter measurements. For the 79 plots included in this analysis, 24% had at least one hollow piece of DCWD. Including measurements of hollowness decreased the DCWD volume on each plot by a maximum of 8.8 m^3^/ha (8.5%) and carbon storage by a maximum of 0.9 Mg C/ha (6.2%). In plots with hollow pieces of DCWD, the mean reduction was 3.2% and 2.6%, respectively. However, since most plots did not have hollow pieces of DCWD, ignoring hollowness reduced volume and carbon storage across all plots by only 0.8% and 0.6%, respectively.

**Figure 6:**
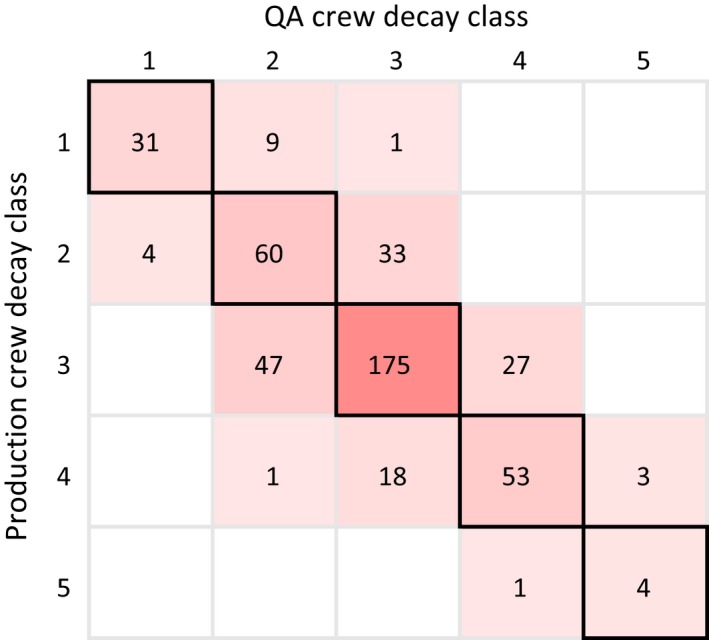
Heat map showing a comparison of the downed coarse woody debris decay class determinations by the QA crew vs. the production crew. Black outlines around cells indicate agreement between the QA and production crews. The value in each cell is the number of pieces of downed coarse woody debris.

Decay class rankings by the production crew for the inventories from 2012–2015 showed that one‐half of all DCWD pieces (52%) were in decay class 3, with most of the remainder in decay classes 4 (23%) and 2 (17%; Table 2). Only a small fraction of DCWD pieces were classified as decay class 1 (7%) or 5 (2%). We compared decay class rankings by QA and production crews for the 467 pieces of DCWD for 2009–2015 (Fig. 6). Results showed that the production crew identified the same decay class as the QA crew for 89% of DCWD pieces for decay class 1, 51% for decay class 2, 77% for decay class 3, 65% for decay class 4, and 57% for decay class 5.

The production crew identified 47 unique tree species for the inventories from 2012–2015. Of all individual pieces of DCWD, 73% were hardwood and 27% were softwood. Production crews identified 76% to the species level and an additional 3% to the genus level only. The remaining pieces were grouped into broader categories, including unknown hardwood (18%), unknown softwood (2%), and unknown (1%). The QA crews were able to identify a greater proportion of DCWD pieces to the species level (82%) and had fewer in the broader categories (11% unknown hardwood, <1% unknown softwood, and <1% unknown). The production crew correctly identified 79% of DCWD pieces to the species level, 86% to the genus level, and 98% as hardwood/softwood, assuming that the QA crew was correct.

**Figure 7:**
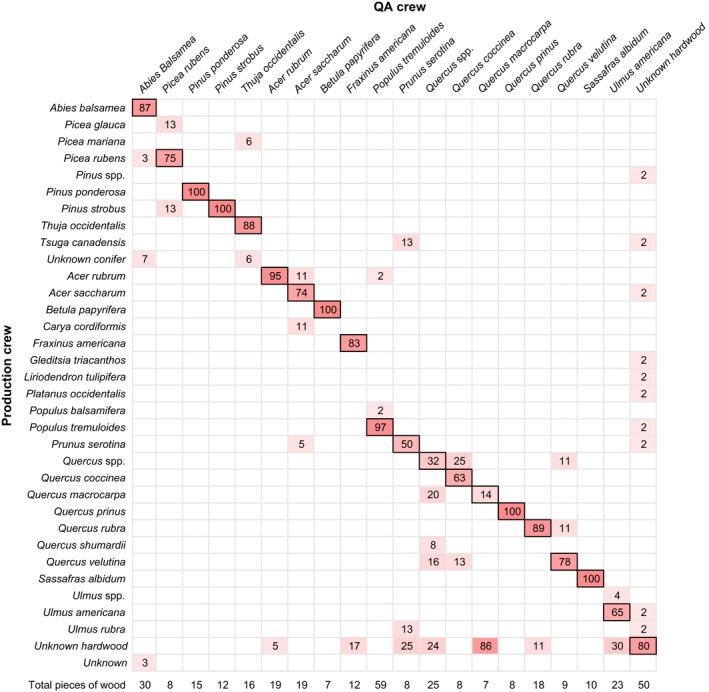
Heat map showing the percentage of pieces of downed coarse woody debris that were identified correctly by the production crew for the 20 most common species identified by the QA crew. Black outlines around cells indicate agreement between the QA and production crews. The total number of pieces of downed coarse woody debris used to calculate the percentage is indicated in the bottom row.

We compared the 20 most common species identified by the QA crew with the production crew identifications and found 100% agreement for five species (*Pinus ponderosa* [ponderosa pine], *Pinus strobus* [white pine], *Betula papyrifera* [paper birch], *Quercus prinus* [chestnut oak], and *Sassafras albidum* [sassafras]; Fig. 7). Other species were more difficult to identify, having lower agreement between production and QA crews, the worst being *Quercus macrocarpa* (bur oak; 14%), *Prunus serotina* (black cherry; 50%), *Quercus coccinea* (scarlet oak; 63%), and *Ulmus americana* (American elm; 65%). For the 23 species found only once by the QA crew, the production crew identified 74% correctly to the species level and 89% to the genus.

Not surprisingly, the degree of decay influenced the ability to identify DCWD. At the species level, 95% of pieces were identified correctly in decay class 1, 89% in decay class 2, 73% in decay class 3, and 74% in decay class 4. There were only 11 pieces of DCWD in decay class 5, and none of them were identified by both the production and QA crews; species identification of highly decomposed wood is challenging.

**Figure 8:**
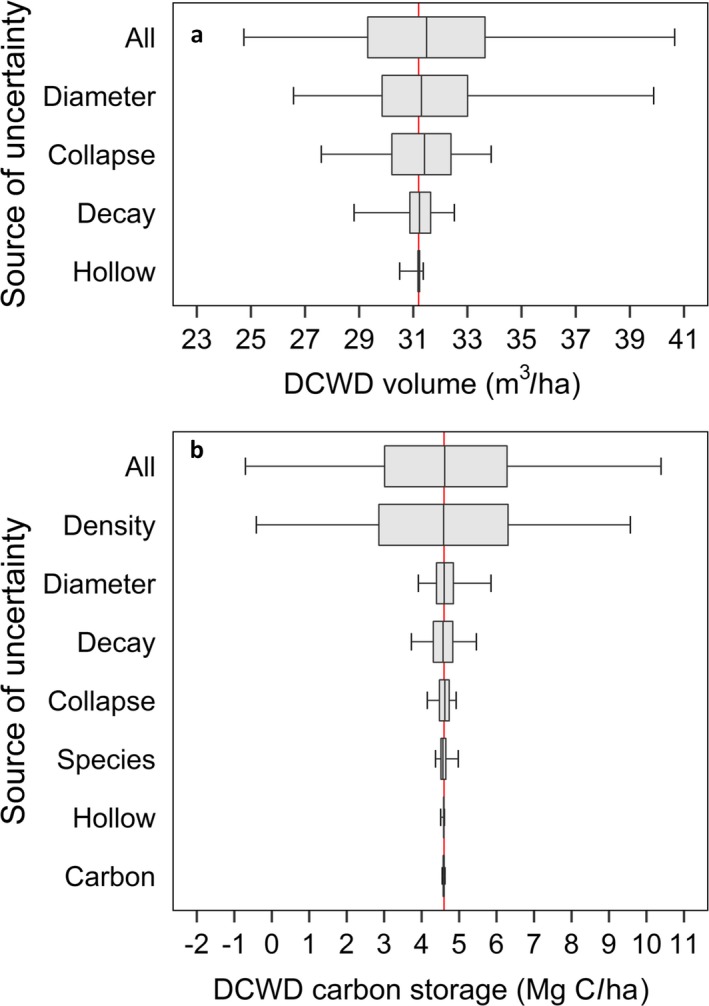
Contribution of each source of uncertainty to downed coarse woody debris (a) volume and (b) carbon storage estimate, and all sources of uncertainty combined. The red line indicates the mean for the 79 plots without uncertainty and the boxplots indicate the uncertainty in the mean values based on the Monte Carlo analysis. The box represents the interquartile range and the whiskers are the 95% confidence interval.

Assessments of uncertainty from the Monte Carlo analysis showed that when all the sources of uncertainty were combined, the average interquartile range was 4.3 m^3^/ha for DCWD volume (Fig. 8, Data S2). The 95% CI, calculated as the difference between 2.5 and 97.5 percentiles of the distribution and taken as our metric of uncertainty, was 15.9 m^3^/ha (Fig. 8a). The greatest source of uncertainty in the DCWD volume estimate was diameter measurement (95% CI = 13.3 m^3^/ha), followed by collapse ratio (95% CI = 6.2 m^3^/ha), decay class (95% CI = 3.7 m^3^/ha) and hollowness (95% CI = 0.9 m^3^/ha). Note that the decay class selection is used to determine the collapse ratio and therefore indirectly affects the estimate of uncertainty in the volume.

For DCWD carbon storage (averaging 4.6 Mg C/ha), the average interquartile range was 3.3 Mg C/ha and the 95% CI was 11.1 Mg C/ha (Fig. 8b). Estimating the density of wood (which is not measured by FIA protocol) was the greatest source of uncertainty. The uncertainty due to density measurement (95% CI = 10 Mg C/ha) was more than five times greater than the uncertainty due to diameter measurement (95% CI = 1.9 Mg C/ha), which was the next greatest source of uncertainty, followed by decay class (95% CI = 1.7 Mg C/ha), collapse ratio (95% CI = 0.8 Mg C/ha), and species identification (95% CI = 0.6 Mg C/ha). The uncertainties due to hollowness (95% CI = 0.10 Mg C/ha) and carbon measurement (95% CI = 0.07 Mg C/ha) were negligible.

## Discussion

Uncertainty analyses help to establish confidence limits, which aid in the interpretation of an estimate. The overall uncertainty in the estimate of DCWD carbon storage was nearly 2.5 times the mean of the 79 plots in the study (4.6 Mg C/ha), indicating a poor ability to quantify this carbon pool. In comparison, the uncertainty estimate for DCWD volume was modest, amounting to about one‐half of the mean (31.2 m^3^/ha).

It can be difficult to compare estimates of DCWD among studies because sampling protocols and definitions for determining what constitutes DCWD vary, such as minimum diameter and length requirements. However, the range in DCWD volume (1–205 m^3^/ha) and carbon (0.1–40 Mg C/ha) across the 79 plots in this study encompassed the range of previously reported values from the region (10–80 m^3^/ha for volume and 2–20 Mg C/ha for carbon storage; Currie and Nadelhoffer 2002, Gough et al. 2007, Bradford et al. 2009), which covered smaller spatial scales.

The amount of DCWD in forests is influenced by multiple factors, such as forest type, stand age, disturbance history, and climate (Harmon et al. 1986). Some of the plots from our analysis that had the highest amounts of DCWD had been recently cut, which can add considerable amounts of DCWD in the form of residual slash. The DCWD volumes for the northeastern United States are generally lower than estimates from some other regions, such as wet tropical forests that have up to 240 m^3^/ha of DCWD volume and 50 Mg C/ha of carbon storage (Clark et al. 2002, Keller et al. 2004, Iwashita et al. 2013). Estimates of DCWD in cool, moist, old‐growth forests of the Pacific Northwest are among the highest reported, reaching DCWD volumes of nearly 350 m^3^/ha (Spies et al. 1988) and 90 Mg C/ha of carbon storage (Smithwick et al. 2002). Old growth forests in the northeastern United States have DCWD values in the range of 26–224 m^3^/ha for volume (McGee et al. 1999, Spetich et al. 1999, Fraver and White 2005, D'Amato et al. 2008, Wesely et al. 2018) and 3–27 Mg C/ha for carbon storage (Fisk et al. 2002, Hoover et al. 2012, Ford and Keeton 2017), but old growth forests are uncommon because much of the region has been harvested at least once.

Our study identified the major sources of uncertainty in measurements of DCWD at the plot level. In comparison, across the landscape, the sampling error, or spatial variability, of DCWD volume and carbon storage for these 79 plots was 16.1 m^3^/ha and 2.8 Mg C/ha, respectively. The uncertainty in measurements propagated in our Monte Carlo analysis was similar to the sampling error for volume (15.9 m^3^/ha), but more than fourfold the sampling error of carbon storage (11.1 Mg C/ha). The FIA database for the region includes 6,095 plots of which only 79 were revisited by QA crews. Future QA inventories on additional plots will enable better characterization of uncertainty due to spatial variation. Clearly, the additional uncertainty incurred in making estimates at the plot scale, especially in carbon storage, is large and in need of attention.

The overall estimate of uncertainty is less than the sum of individual sources of uncertainty. If errors are independent and random, they can be combined by calculating the root sum of squares, which is less computationally demanding than the Monte Carlo method we used. We compared the overall uncertainty calculated with the Monte Carlo method with a combined uncertainty calculated by summing the individual errors in quadrature, and found them to be in good agreement (15.2 vs. 15.9 m^3^/ha for volume and 10.4 vs. 11.1 Mg C/ha for carbon storage) in spite of the non‐normal distributions of some of the error sources (Fig. 8).

Estimating the density of wood was the greatest single source of uncertainty in DCWD carbon storage, contributing 66% of the sum of all individual sources. Wood density is not measured in FIA inventories, but estimates of density are needed for converting DCWD volume to mass. Wood density varies substantially by species and decay class, and measurements at this level are scarce and come from many locations (see Data S1). Although density decreased with decay class, there was not a clear increase in variability with level of decay when all species are combined (Fig. 3b), which reflects different rates of density loss with decay class by species (Harmon et al. 2008).

For the tree species included in this analysis, 31% of the density values across decay classes were based on measurements of the individual species, 40% on values from other species within the genus, and 29% on other genera because neither the species nor genus were sampled. Harmon et al. (2008) determined that characterizing density for a genus reduced uncertainty by up to 50%, compared to using estimates for other genera. Substantial variability in density can occur even within a species and decay class, especially when logs are in advanced stages of decay but still have relatively undecayed and dense heartwood (Brown 2002).

Because published density data come from diverse regions and are obtained with different methods, applying these data results in higher uncertainty than would be obtained by sampling from the population of inference. Density measurements for each individual piece of DCWD would be ideal, but collecting and processing wood samples for this purpose on broad scales is prohibitively laborious and costly. More rapid, non‐destructive, field methods for measuring the density of standing trees are becoming increasingly prevalent and reliable (Gao et al. 2017), although these methods have not been widely applied to DCWD density measurement, and would need refinement before being adopted (e.g., Kahl et al. 2009, Mäkipää and Linkosalo 2011). By whatever means, better estimates of density are essential to reduce uncertainty in DCWD mass or carbon storage (Brown 2002). A more comprehensive and systematic sampling of DCWD density would improve estimates of carbon storage in forests and could potentially be implemented as part of routine FIA field data collection procedures once the methods for measuring DCWD density have reached an acceptable level of efficiency and precision.

Because estimates of DCWD volume do not require knowledge of wood density, they are much more reliable than those of carbon storage. In studies of forest biodiversity, DCWD volume is the metric of choice (Stokland et al. 2012), in part because of the strong link between DCWD volume and the richness of deadwood‐dependent organisms (Lassauce et al. 2011).

Diameter measurement error was the greatest source of uncertainty for DCWD volume and the second greatest source for carbon storage. Uncertainty in the diameter of DCWD is greater than uncertainty in diameter measurements of standing trees. We evaluated standing live tree data from FIA plots using the same approach as for DCWD and determined that the measurement uncertainty in diameter at breast height was 0.14 cm, or 0.7% of the mean tree diameter, whereas the DCWD measurement error was 1.0 cm or about 7% of the mean DCWD diameter. Measuring the diameter of DCWD is inherently difficult for many reasons, such as identifying the point to be measured given the different orientations of DCWD pieces along the transect, various shapes of log cross‐sections, poorly defined edges of decayed logs, and the inability to measure the circumference of DCWD that is on the ground. Many of these challenges may be insurmountable, but using calipers would likely improve diameter measurements compared to holding a ruler above the log.

The decay class is used to determine the density and the collapse ratio. The high agreement between the production and QA crews for decay class 1 indicates that the least decayed pieces of DCWD are the easiest to classify. More DCWD is commonly found in decay class 3, suggesting that the residence time of logs is longest in this decay class (Harmon et al. 1986), and this was true in our data set (Fig. 6), but we found that the production crew tended to identify more DCWD in this decay class than did the QA crew. The FIA minimum acceptable standard for decay class determination is plus or minus one decay class for 90% of the comparisons (Westfall and Woodall 2007). If individuals on the production crew are off by more than one decay class, it impacts their job performance evaluation, which may explain why production crews tend to bias their classifications toward more central values. Specifically, for logs identified as decay class 2 and 4 by the QA crew, there was greater tendency by the production crew to select decay class 3 than either 1 or 5 (Fig. 6) because they would be less likely to be off by more than 1 decay class.

The collapse ratio values used in this analysis were from a case study by Fraver et al. (2013) with a relatively small sample size (125 samples across all decay classes). The collapse ratio uncertainty could be reduced by collecting or compiling more data across decay classes and species to better characterize collapse. Currently, FIA does not account for DCWD collapse in its biomass calculations (i.e., in all stages of decay they measure only the width of logs and do not account for changes in height), which, according to our results, leads to an overestimation of mean DCWD volume by 9% (33% maximum) and carbon storage by 7% (maximum of 30%) across plots. Thus, accounting for collapse would reduce bias in DCWD estimates.

Tree species identification contributed relatively little to the overall uncertainty in carbon storage in part because agreement was high between production and QA crews (Fig. 7). Tree species identification, in combination with decay class, is used to determine DCWD density, but taxonomic class is not as important as decay class in predicting variability in density (Fig. 8, Data S1). Tree species (hardwood vs. softwood) is also used to estimate carbon concentrations, but this was a negligible source of uncertainty.

The hollowness of DCWD pieces is included in FIA DCWD volume calculations, but our analyses suggest it is less important to include than the collapse ratio. Despite the potential challenges in measuring hollowness because of the odd shape of cavities and difficulties determining whether a log is hollow where it intersects the transect, the estimates of hollowness were highly reproducible. However, accounting for hollowness had little effect on DCWD estimates because so few logs were recorded as hollow. The inclusion of hollowness may be important in other regions, but in the northeastern United States, cavities are typically small and uncommon. Unlike the collapse ratio, which can be estimated from the decay class, quantifying hollowness requires costly and time‐consuming field measurements of individual DCWD pieces. Based on our analyses, if FIA were to eliminate this procedure, it would have little impact on estimates of DCWD in this region.

The carbon concentration was the least important source of uncertainty evaluated for carbon storage. The standard errors for both hardwoods and softwoods were small (less than 0.05%). Additional measurements of the carbon concentration of DCWD would do little to reduce the uncertainty and are not needed. Although estimates of DCWD mass are often reported in the literature, we did not report them here because our analyses show that carbon concentrations were always close to 50% and a negligible source of uncertainty.

Although some sources of uncertainty in this analysis, such as carbon concentration, were minor and could have been omitted without any substantial influence on the results, it is important to recognize that other sources of uncertainty could have been included if additional data were available. We have no data on the rate at which pieces of DCWD are overlooked by one crew but not the other (Jordan et al. 2004) because individual pieces were not tagged in the field. This is a source of error that could be quantified using a different approach to remeasurement. Other examples of error sources not reported here include uncertainty due to the inventory method selected (e.g., line‐intersect, fixed‐area, point relascope; Jordan et al. 2004), data sets selected for the Monte Carlo analysis (i.e., collapse ratio, density, and carbon concentration), and errors in the delineation of plot boundaries or transect lines.

Data entry errors can also contribute to uncertainty. Although the FIA data used in our analysis had undergone quality control procedures, two suspect values became evident during the comparison between the production and QA crews. In one case for a plot in Illinois, the QA crew listed a species code of 988 (*Laguncularia racemosa* [white mangrove]) for a piece of DCWD that the production crew identified as 998 (unknown hardwood). The former species code is likely a data entry error since the species codes are similar and white mangrove is a coastal species not found in Illinois. Including this erroneous species code in the Monte Carlo had no impact because the same initial density and reduction factors are used for both white mangrove and unknown hardwood. In another case, the production and QA crews recorded extremely different diameter measurements (152.4 vs. 12.7 cm) for the same piece of DCWD. The larger measurement was greater than any other diameter measured by nearly 100 cm and therefore was almost certainly a recording error. We decided to exclude this case from the Monte Carlo analysis because it did not represent the type of error we were interested in quantifying.

Finally, our analysis focused on the USDA Forest Service FIA data set for the northeastern United States, which allowed us to evaluate uncertainty in a set of parameters particular to that protocol. The extent to which our general findings apply to other approaches to measuring DCWD remains unknown. For example, variability in DCWD volume estimates is known to decrease with increasing line‐intersect transect length (Woldendorp et al. 2004); however, the fixed transect length used by FIA did not allow us to address this variable. Further, for protocols using fixed‐area plots, where all DCWD pieces within plot boundaries are inventoried, additional sources of uncertainty arise. These include decisions regarding the treatment of pieces crossing the plot boundary (Gove and Van Deusen 2011) and bias resulting from assumptions of DCWD piece shape, which can produce per‐unit‐area estimates that differ by as much as 38% depending on which shape (and associated volume formula) is assumed (Fraver et al. 2007).

## Conclusions

Knowledge of the uncertainty in DCWD estimates is critically important for understanding and interpreting DCWD data used in applications such as national greenhouse gas inventories, biodiversity assessments, and fuel loading estimates for determining risk of wildland fire. Results of our analysis using FIA data from the northeastern US indicate that the uncertainty in estimates of DCWD carbon storage is large, mostly due to uncertainty in the density of DCWD. As time and technology permit, including field measurements of density in the FIA protocol has the potential to substantially improve confidence in the values. Volume estimates are not subject to this source of uncertainty.

Estimates of both DCWD carbon storage and volume could be improved by including the collapse ratio in DCWD calculations. In this analysis, we used collapse ratios reported from another study (Fraver et al. 2013). The uncertainty in this data set of collapse ratios could be reduced through additional sampling across decay classes and a broader spectrum of species. Alternatively, the height of individual pieces of DCWD could be measured in the field, which would obviate the need for the use of collapse ratios by decay class and would improve estimates of DCWD volume. However, this would be a time‐consuming measurement and destructive for partially buried logs.

Additional steps could be taken to reduce measurement errors in the field. Although FIA technicians receive substantial training, DCWD inventories could benefit from greater objectivity and uniformity in measurement procedures. For example, identification of DCWD species and decay classes could be improved with training aids, such as keys and photographs.

Some sources of uncertainty, such as carbon concentration, are already well characterized; therefore, additional measurements are not needed because they would not reduce the uncertainty. Sampling efficiency could be improved by eliminating measurement of hollow logs, since it has little effect on estimates of DCWD. Although eliminating measurements of hollowness could save time with little impact on estimates of the amount of carbon stored in DCWD, hollowness may be important for other applications such as evaluating wildlife habitat. Lastly, it is important to recognize the value of blind QA measurements that make analyses of uncertainty possible. While time consuming and costly, these measurements add great value to the data because they can be used to determine the reliability of estimates so that results from field inventories can be evaluated in a broader context.

## Notes

1 https://apps.fs.usda.gov/fia/datamart/CSV/datamart_csv.html

2 https://www.fia.fs.fed.us/library/index.php

## References

[eap1844-bib-0001] Bradford, J. , P. Weishampel , M.‐L. Smith , R. Kolka , R. A. Birdsey , S. V. Ollinger , and M. G. Ryan . 2009 Detrital carbon pools in temperate forests: magnitude and potential for landscape‐scale assessment. Canadian Journal of Forest Research 39:802–813.

[eap1844-bib-0002] Brand, G. J. , M. D. Nelson , D. G. Wendt , and K. K. Nimerfro . 2000 The hexagon/panel system for selecting FIA plots under an annual inventory Pages 8–13 *in* McRoberts R. E., Reams G. A., and Van Deusen P. C., editors. Proceedings of the first annual forest inventory and analysis symposium, General Technical Report NC‐213. U.S. Department of Agriculture, Forest Service, North Central Research Station, St. Paul, Minnesota, USA.

[eap1844-bib-0003] Brown, S. L. 2002 Measuring carbon in forests: current status and future challenges. Environmental Pollution 116:363–372. 11822714 10.1016/s0269-7491(01)00212-3

[eap1844-bib-0004] Clark, D. B. , D. A. Clark , S. Brown , S. F. Oberbauer , and E. Veldkamp . 2002 Stocks and flows of coarse woody debris across a tropical rain forest nutrient and topography gradient. Forest Ecology and Management 164:237–248.

[eap1844-bib-0005] Cobb, R. C. , M. N. Chan , R. K. Meentemeyer , and D. M. Rizzo . 2012 Common factors drive disease and coarse woody debris dynamics in forests impacted by sudden oak death. Ecosystems 15:242–255.

[eap1844-bib-0006] Currie, W. S. , and K. J. Nadelhoffer . 2002 The imprint of land‐use history: Patterns of carbon and nitrogen in downed woody debris at the Harvard Forest. Ecosystems 5:446–460.

[eap1844-bib-0007] Dale, V. H. , et al. 2001 Climate change and forest disturbances. BioScience 51:723–734.

[eap1844-bib-0008] D'Amato, A. W. , D. A. Orwig , and D. R. Foster . 2008 The influence of successional processes and disturbance on the structure of *Tsuga canadensis* forests. Ecological Applications 18:1182–1199. 18686580 10.1890/07-0919.1

[eap1844-bib-0009] Domke, G. M. , C. W. Woodall , B. F. Walters , and J. E. Smith . 2013 From models to measurements: comparing down dead wood carbon stock estimates in the U.S. forest inventory. PLoS ONE 8:e59949. 23544112 10.1371/journal.pone.0059949 PMC3609740

[eap1844-bib-0068] Fahey, T. J. , et al. 2005 The biogeochemistry of carbon at Hubbard Brook. Biogeochemistry 75:109–179.

[eap1844-bib-0010] Fisk, M. C. , D. R. Zak , and T. R. Crow . 2002 Nitrogen storage and cycling in old‐ and second‐growth northern hardwood forests. Ecology 83:73–87.

[eap1844-bib-0011] Ford, S. E. , and W. S. Keeton . 2017 Enhanced carbon storage through management for old‐growth characteristics in northern hardwood‐conifer forests. Ecosphere 8:e01721.

[eap1844-bib-0012] Fraver, S. , and A. S. White . 2005 Disturbance dynamics of old‐growth *Picea rubens* forests of northern Maine. Journal of Vegetation Science 16:597–610.

[eap1844-bib-0013] Fraver, S. , R. G. Wagner , and M. Day . 2002 Dynamics of coarse woody debris following gap harvesting in the Acadian forest of central Maine, U.S.A. Canadian Journal of Forest Research 32:2094–2105.

[eap1844-bib-0014] Fraver, S. , A. Ringvall , and B. G. Jonsson . 2007 Refining volume estimates of down woody debris. Canadian Journal of Forest Research 37:627–633.

[eap1844-bib-0015] Fraver, S. , A. M. Milo , J. B. Bradford , A. W. D'Amato , L. Kenefic , B. J. Palik , C. W. Woodall , and J. Brissette . 2013 Woody debris volume depletion through decay: Implications for biomass and carbon accounting. Ecosystems 16:1262–1272.

[eap1844-bib-0016] Freedman, B. , V. Zelazny , D. Beaudette , T. Fleming , G. Johnson , S. Flemming , J. S. Gerrow , G. Forbes , and S. Woodley . 1996 Biodiversity implications of changes in the quantity of dead organic matter in managed forests. Environmental Reviews 4:238–265.

[eap1844-bib-0017] Gao, S. , X. Wang , M. C. Wiemann , B. K. Brashaw , R. J. Ross , and L. Wang . 2017 A critical analysis of methods for rapid and nondestructive determination of wood density in standing trees. Annals of Forest Science 74:1–13.

[eap1844-bib-0018] Goldin, S. R. , and M. F. Hutchinson . 2014 Coarse woody debris reduces the rate of moisture loss from surface soils of cleared temperate Australian woodlands. Soil Research 52:637–644.

[eap1844-bib-0019] Gough, C. M. , C. S. Vogel , C. Kazanski , L. Nagel , C. E. Flower , and P. S. Curtis . 2007 Coarse woody debris and the carbon balance of a north temperate forest. Forest Ecology and Management 244:60–67.

[eap1844-bib-0020] Gove, J. H. , and P. C. Van Deusen . 2011 On fixed‐area plot sampling for downed coarse woody debris. Forestry 84:109–117.

[eap1844-bib-0021] Gurnell, A. M. , K. J. Gregory , and G. E. Petts . 1995 The role of coarse woody debris in forest aquatic habitats: Implications for management. Aquatic Conservation: Marine and Freshwater Ecosystems 5:143–166.

[eap1844-bib-0022] Hagan, J. M. , and S. L. Grove . 1999 Coarse woody debris: Humans and nature competing for trees. Journal of Forestry 97:6–11.

[eap1844-bib-0023] Harmon, M. E. , and J. F. Franklin . 1989 Tree seedlings on logs in *Picae*‐*Tsuga* forests of Oregon and Washington. Ecology 70:48–59.

[eap1844-bib-0024] Harmon, M. E. , and C. Hua . 1991 Coarse woody debris dynamics in two old‐growth ecosystems. BioScience 41:604–610.

[eap1844-bib-0025] Harmon, M. E. , and J. Sexton . 1995 Water balance of conifer logs in early stages of decomposition. Plant and Soil 172:141–152.

[eap1844-bib-0026] Harmon, M. E. , et al. 1986 Ecology of coarse woody debris in temperate ecosystems. Advances in Ecological Research 15:133–302.

[eap1844-bib-0027] Harmon, M. E. , C. W. Woodall , B. Fasth , and J. Sexton . 2008 Woody detritus density and density reduction factors for tree species in the United States: a synthesis. General Technical Report NRS‐29. U.S. Department of Agriculture, Forest Service, Northern Research Station, Newtown Square, Pennsylvania, USA.

[eap1844-bib-0028] Hoover, C. M. , W. B. Leak , and B. G. Keel . 2012 Benchmark carbon stocks from old‐growth forests in northern New England, USA. Forest Ecology and Management 266:108–114.

[eap1844-bib-0029] Iwashita, D. K. , C. M. Litton , and C. P. Giardina . 2013 Coarse woody debris carbon storage across a mean annual temperature gradient in tropical montane wet forest. Forest Ecology and Management 291:336–343.

[eap1844-bib-0030] Janowiak, M. K. , and C. R. Webster . 2010 Promoting ecological sustainability in woody biomass harvesting. Journal of Forestry 108:16–23.

[eap1844-bib-0031] Jordan, G. J. , M. J. Ducey , and J. H. Gove . 2004 Comparing line‐intersect, fixed‐area, and point relascope sampling for dead and downed coarse woody material in a managed northern hardwood forest. Canadian Journal of Forest Research 34:1766–1775.

[eap1844-bib-0032] Kahl, T. , C. Wirth , M. Mund , G. Böhnisch , and E.‐D. Schulze . 2009 Using drill resistance to quantify the density in coarse woody debris of Norway spruce. European Journal of Forest Research 128:467–473.

[eap1844-bib-0033] Kaiser, L. 1983 Unbiased estimation in line‐interception sampling. Biometrics 39:965–976.

[eap1844-bib-0034] Keller, M. , M. Palace , G. P. Asner , R. Pereira , and J. N. M. Silva . 2004 Coarse woody debris in undisturbed and logged forests in the eastern Brazilian Amazon. Global Change Biology 10:784–795.

[eap1844-bib-0069] Lassauce, A. , Y. Paillet , H. Jactel , and C. Bouget . 2011 Deadwood as a surrogate for forest biodiversity: Meta‐analysis of correlations between deadwood volume and species richness of saproxylic organisms. Ecological Indicators 11:1027–1039.

[eap1844-bib-0035] Magnússon, R. Í. , A. Tietema , J. H. C. Cornelissen , M. M. Hefting , and K. Kalbitz . 2016 Tamm review: Sequestration of carbon from coarse woody debris in forest soils. Forest Ecology and Management 377:1–15.

[eap1844-bib-0036] Mäkipää, R. , and T. Linkosalo . 2011 A non‐destructive field method for measuring wood density of decaying logs. Silva Fennica 45:1135–1142.

[eap1844-bib-0037] Maser, C. A. , R. G. Anderson , K. Cromack Jr. , J. T. Williams , and R. E. Martin . 1979 Dead and down woody material Pages 78–95 *in* Thomas J. W., editor. Wildlife habitats in managed forests: The Blue Mountains of Oregon and Washington. Agriculture Handbook 553. USDA Forest Service, Washington, D.C., USA.

[eap1844-bib-0038] McCarthy, B. C. , and R. R. Bailey . 1994 Distribution and abundance of coarse woody debris in a managed forest landscape of the central Appalachians. Canadian Journal of Forest Research 24:1317–1329.

[eap1844-bib-0039] McComb, W. , and D. B. Lindenmayer . 1999 Dying, dead and down trees Pages 335–372 *in* Hunter M. III, editor. Managing biodiversity in forest ecosystems. Cambridge University Press, New York, New York, USA.

[eap1844-bib-0040] McGee, G. G. , D. J. Leopold , and R. D. Nyland . 1999 Structural characteristics of old‐growth, maturing, and partially cut northern hardwood forests. Ecological Applications 9:1316–1329.

[eap1844-bib-0041] Oswalt, S. N. , W. B. Smith , P. D. Miles , and S. A. Pugh . 2014 Forest resources of the United States, 2012: a technical document supporting the Forest Service 2010 update of the RPA assessment. General Technical Report WO‐91. U.S. Department of Agriculture, Forest Service, Washington Office, Washington, D.C., USA.

[eap1844-bib-0042] Pan, Y. , et al. 2011 A large and persistent carbon sink in the world's forests. Science 333:988–993. 21764754 10.1126/science.1201609

[eap1844-bib-0043] Pedlar, J. H. , J. L. Pearce , L. A. Venier , and D. W. McKenney . 2002 Coarse woody debris in relation to disturbance and forest type in boreal Canada. Forest Ecology and Management 158:189–194.

[eap1844-bib-0070] R Core Team . 2017 R: A language and environment for statistical computing. R Foundation for Statistical Computing, Vienna, Austria https://www.R-project.org/

[eap1844-bib-0044] Riffell, S. , J. Verschuyl , D. Miller , and T. B. Wigley . 2011 Biofuel harvests, coarse woody debris, and biodiversity—a meta‐analysis. Forest Ecology and Management 261:878–887.

[eap1844-bib-0045] Schoennagel, T. , T. T. Veblen , and W. H. Romme . 2004 The interaction of fire, fuels, and climate across Rocky Mountain forests. BioScience 54:661–676.

[eap1844-bib-0046] Shortle, W. C. , K. T. Smith , J. Jellison , and J. S. Schilling . 2012 Potential of decaying wood to restore root‐available base cations in depleted forest soils. Canadian Journal of Forest Research 42:1015–1024.

[eap1844-bib-0047] Siitonen, J. 2001 Forest management, coarse woody debris and saproxylic organisms: Fennoscandian boreal forests as an example. Ecological Bulletins 49:11–41.

[eap1844-bib-0048] Siitonen, J. , P. Martikainen , P. Punttila , and J. Rauh . 2000 Coarse woody debris and stand characteristics in mature managed and old‐growth boreal mesic forests in southern Finland. Forest Ecology and Management 128:211–225.

[eap1844-bib-0049] Smithwick, E. A. H. , M. E. Harmon , S. M. Remillard , S. A. Acker , and J. F. Franklin . 2002 Potential upper bounds of carbon stores in forests of the Pacific Northwest. Ecological Applications 12:1303–1317.

[eap1844-bib-0050] Sollins, P. 1982 Input and decay of coarse woody debris in coniferous stands in western Oregon and Washington. Canadian Journal of Forest Research 12:18–28.

[eap1844-bib-0051] Spetich, M. A. , S. R. Shifley , and G. R. Parker . 1999 Regional distribution and dynamics of coarse woody debris in Midwestern old‐growth forests. Forest Science 45:302–313.

[eap1844-bib-0052] Spies, T. A. , J. F. Franklin , and T. B. Thomas . 1988 Coarse woody debris in Douglas‐Fir forests of western Oregon and Washington. Ecology 69:1689–1702.

[eap1844-bib-0053] Stokland, J. N. , J. Siitonen , and B. G. Jonsson . 2012 Biodiversity in dead wood. Cambridge University Press, New York, New York, USA.

[eap1844-bib-0054] Sturtevant, B. R. , J. A. Bissonette , J. N. Long , and D. W. Roberts . 1997 Coarse woody debris as a function of age, stand structure, and disturbance in boreal Newfoundland. Ecological Applications 7:702–712.

[eap1844-bib-0055] Ucitel, D. , D. P. Christian , and J. M. Graham . 2003 Vole use of coarse woody debris and implications for habitat and fuel management. Journal of Wildlife Management 67:65–72.

[eap1844-bib-0056] USDA Forest Service . 2017 Forest inventory and analysis national core field guide. Volume 1: Field data collection procedures for Phase 2 plots. Version 7.2. U.S. Department of Agriculture, Forest Service, Washington, D.C., USA https://www.fia.fs.fed.us/library/field-guides-methods-proc/

[eap1844-bib-0057] Warren, W. G. , and P. F. Olsen . 1964 A line intersect technique for assessing logging waste. Forest Science 13:267–276.

[eap1844-bib-0058] Wesely, N. , S. Fraver , L. Kenefic , A. Weiskittel , J.‐C. Ruel , M. Thompson , and A. White . 2018 Structural attributes of old‐growth and partially harvested northern white‐cedar stands in northeastern North America. Forests 9:376.

[eap1844-bib-0059] Westfall, J. A. , and C. W. Woodall . 2007 Measurement repeatability of a large‐scale inventory of forest fuels. Forest Ecology and Management 253:171–176.

[eap1844-bib-0060] Woldendorp, G. , R. J. Keenan , S. Barry , and R. D. Spencer . 2004 Analysis of sampling methods for coarse woody debris. Forest Ecology and Management 198:133–148.

[eap1844-bib-0061] Woodall, C. W. , and V. J. Monleon . 2008 Sampling protocol, estimation, and analysis procedures for the down woody materials indicator of the FIA program. General Technical Report NRS‐22. U.S. Department of Agriculture, Forest Service, Northern Research Station, Newtown Square, Pennsylvania, USA.

[eap1844-bib-0062] Woodall, C. W. , L. S. Heath , and J. E. Smith . 2008 National inventories of down and dead woody material forest carbon stocks in the United States: Challenges and opportunities. Forest Ecology and Management 256:221–228.

[eap1844-bib-0063] Woodall, C. W. , B. F. Walters , and J. A. Westfall . 2012 Tracking downed dead wood in forests over time: Development of a piece matching algorithm for line intercept sampling. Forest Ecology and Management 277:196–204.

[eap1844-bib-0064] Woodall, C. W. , B. F. Walters , S. N. Oswalt , G. M. Domke , C. Toney , and A. N. Gray . 2013 Biomass and carbon attributes of downed woody materials in forests of the United States. Forest Ecology and Management 305:48–59.

[eap1844-bib-0065] Woodall, C. W. , M. B. Russell , B. F. Walters , A. W. D'Amato , S. Fraver , and G. M. Domke . 2015 Net carbon flux of dead wood in forests of the eastern US. Oecologia 177:861–874. 25430045 10.1007/s00442-014-3171-8

[eap1844-bib-0066] Yuan, J. , L. Hou , X. Wei , Z. Shang , F. Cheng , and S. Zhang . 2017 Decay and nutrient dynamics of coarse woody debris in the Qinling Mountains, China. PLoS ONE 12:e0175203. 28384317 10.1371/journal.pone.0175203 PMC5383274

[eap1844-bib-0067] Zhu, J. , et al. 2017 Carbon stocks and changes of dead organic matter in China's forests. Nature Communications 8:151. 10.1038/s41467-017-00207-1 PMC5532249 28751686

