# Supplementary material for: Estimating uncertainty in the volume and carbon storage of downed coarse woody debris
Source: Ecol Appl. 2019 Jan 28;29(2):e01844. doi: 10.1002/eap.1844 (PMC6850466; doi:10.1002/eap.1844)
Supplement: Supplementary file 1 [file EAP-29-na-s001.pdf]

John L. Campbell, Mark B. Green, Ruth D. Yanai, Christopher W. Woodall, Shawn Fraver, Mark E. Harmon, Mark A. Hatfield, Charles J. Barnett, Craig R. See, and Grant M. Domke. Estimating uncertainty in the volume and carbon storage of coarse woody debris. *Ecological Applications*. 2019.

Data S1

R code and data for the Monte Carlo analysis that quantifies uncertainty in estimates of downed coarse woody debris

---

Authors

John L. Campbell
USDA Forest Service
Northern Research Station
271 Mast Rd.
Durham, NH 03801, USA
Email: jlcampbell@fs.fed.us

Mark B. Green
Center for the Environment
Plymouth State University
17 High Street
Plymouth, NH 03264, USA
Email: mbgreen@plymouth.edu

Ruth D. Yanai
SUNY College of Environmental Science and Forestry
Department of Forest and Natural Resources Management
210 Marshall Hall
1 Forestry Dr.
Syracuse, New York 13210, USA
Email: rdyanai@esf.edu

Christopher W. Woodall
USDA Forest Service
Northern Research Station
271 Mast Rd.
Durham, NH 03801, USA
Email: cwoodall@fs.fed.us

Shawn Fraver
University of Maine
School of Forest Resources
5755 Nutting Hall
Orono, ME 04469, USA
Email: shawn.fraver@maine.edu

Mark E. Harmon
Oregon State University
Forest Ecosystems and Society
210 Richardson Hall
Corvallis, OR, 97331, USA
Email: mark.harmon@oregonstate.edu

Mark A. Hatfield
USDA Forest Service
Northern Research Station
271 Mast Rd.
Durham, NH 03801, USA
Email: mahatfield@fs.fed.us

Charles J. Barnett
USDA Forest Service
Northern Research Station
11 Campus Blvd., Suite 200
Newtown Square, PA, 19073, USA
Email: cjbarnett@fs.fed.us

Craig R. See
University of Minnesota
Department of Ecology, Evolution and Behavior
1987 Upper Buford Circle
St. Paul, MN 55108, USA
Email: crsee@umn.edu

Grant M. Domke
USDA Forest Service
Northern Research Station
1992 Folwell Avenue
St. Paul, MN, 55108, USA
Email: gmdomke@fs.fed.us

File list

dcwd_uncertainty.R

dcwd_collapse_ratio.csv

dcwd_density.csv

fia_dcwd_data.csv

ref_species.csv

Description

dcwd_uncertainty.R – Source code (R v3.4.1) for estimating uncertainty in coarse woody debris using a Monte Carlo approach.

dcwd_collapse_ratio.csv – Data file containing the collapse ratio (height:width) by decay class from Fraver et al. (2013). Collapse ratios are based on data from three species: *Pinus resinosa, Populus tremuloides, and Picea glauca*. Decay class is represented with a 5-class scale, where 1 is the least decomposed and 5 is the most decomposed (USDA Forest Service 2017).

dcwd_density.csv – Data file containing the mean density and density reduction value (decayed density:undecayed density), standard errors, and levels of uncertainty for each decay class (Harmon et al. 2008). "Levels" reflect whether uncertainty was determined for the species (A), was based on the average of species in this genus, because this species was not reported (B), or was based on other species, because this genus was not reported (C).

fia_dcwd_data.csv – Forest Inventory and Analysis quality assurance data. Columns represent the state code (STATECD), county code (COUNTYCD), plot (PLOT_FIADB), inventory year (INVYR), subplot (SUBP), transect (TRANSECT), protocol used by the production crew (PROTOCOL_PROD), protocol used by the QA crew (PROTOCOL_QA), transect diameter measured by the production crew (TRANSDIA_PROD), transect diameter measured by the QA crew (TRANSDIA_QA), species code determined by the production crew (SPCD_PROD), species code determined by the QA crew (SPCD_QA), hollow diameter measured by the production crew (HOLLOW_DIA_PROD), hollow diameter measured by the QA crew (HOLLOW_DIA_QA), decay class determined by the production crew (DECAYCD_PROD), decay class determined by the QA crew (DECAYCD_QA). Additional information about the database is provided by O'Connell et al. (2015).

ref_species.csv – Data file containing the species code, descriptive common name, scientific name, and many other attributes for each tree species. For this application, the most pertinent columns are the carbon concentration (DWM_CARBON_RATIO), the initial density (WOOD_SPRG_GREENVOL_DRYWT), and the softwood/hardwood designation for assigning a carbon concentration (SFTWD_HRDWD). Additional information about the database is provided by O'Connell et al. (2015).

Literature Cited

Fraver, S., A. M. Milo, J. B. Bradford, A. W. D'Amato, L. Kenefic, B. J. Palik, C. W. Woodall, and J. Brissette. 2013. Woody debris volume depletion through decay: Implications for biomass and carbon accounting. Ecosystems **16**:1262-1272.

Harmon, M. E., C. W. Woodall, B. Fasth, and J. Sexton. 2008. Woody detritus density and density reduction factors for tree species in the United States: a synthesis. Gen. Tech. Rep. NRS-29. Newtown Square, PA: U.S. Department of Agriculture, Forest Service, Northern Research Station. 84 p.

O'Connell, Barbara M.; LaPoint, Elizabeth B.; Turner, Jeffery A.; Ridley, Ted; Pugh, Scott A.; Wilson, Andrea M.; Waddell, Karen L.; Conkling, Barbara L. 2015. The Forest Inventory and Analysis Database: Database description and user guide version 6.0.2 for Phase 2. U.S. Department of Agriculture, Forest Service. 748 p. [Online]. Available at web address: http://www.fia.fs.fed.us/library/database-documentation/.

USDA Forest Service. 2017. Forest Inventory and Analysis national core field guide. Volume 1: Field data collection procedures for Phase 2 plots. Version 7.2. Washington, D.C.: U.S. Department of Agriculture, Forest Service. 433 p. https://www.fia.fs.fed.us/library/field-guides-methods-proc/.
