# Supplementary material for: Estimating uncertainty in the volume and carbon storage of downed coarse woody debris
Source: Ecol Appl. 2019 Jan 28;29(2):e01844. doi: 10.1002/eap.1844 (PMC6850466; doi:10.1002/eap.1844)
Supplement: Supplementary file 2 [file EAP-29-na-s002.pdf]

John L. Campbell, Mark B. Green, Ruth D. Yanai, Christopher W. Woodall, Shawn Fraver, Mark E. Harmon, Mark A. Hatfield, Charles J. Barnett, Craig R. See, and Grant M. Domke. Estimating uncertainty in the volume and carbon storage of coarse woody debris. *Ecological Applications*. 2019.

Data S2

Uncertainty values for downed coarse woody debris volume and carbon storage at United States Forest Service Forest Inventory Analysis plots

---

Authors

John L. Campbell
USDA Forest Service
Northern Research Station
271 Mast Rd.
Durham, NH 03801, USA
Email: jlcampbell@fs.fed.us

Mark B. Green
Center for the Environment
Plymouth State University
17 High Street
Plymouth, NH 03264, USA
Email: mbgreen@plymouth.edu

Ruth D. Yanai
SUNY College of Environmental Science and Forestry
Department of Forest and Natural Resources Management
210 Marshall Hall
1 Forestry Dr.
Syracuse, New York 13210, USA
Email: rdyanai@esf.edu

Christopher W. Woodall
USDA Forest Service
Northern Research Station
271 Mast Rd.
Durham, NH 03801, USA
Email: cwoodall@fs.fed.us

Shawn Fraver
University of Maine
School of Forest Resources
5755 Nutting Hall
Orono, ME 04469, USA
Email: shawn.fraver@maine.edu

Mark E. Harmon
Oregon State University
Forest Ecosystems and Society
210 Richardson Hall
Corvallis, OR, 97331, USA
Email: mark.harmon@oregonstate.edu

Mark A. Hatfield
USDA Forest Service
Northern Research Station
271 Mast Rd.
Durham, NH 03801, USA
Email: mahatfield@fs.fed.us

Charles J. Barnett
USDA Forest Service
Northern Research Station
11 Campus Blvd., Suite 200
Newtown Square, PA, 19073, USA
Email: cjbarnett@fs.fed.us

Craig R. See
University of Minnesota
Department of Ecology, Evolution and Behavior
1987 Upper Buford Circle
St. Paul, MN 55108, USA
Email: crsee@umn.edu

Grant M. Domke
USDA Forest Service
Northern Research Station
1992 Folwell Avenue
St. Paul, MN, 55108, USA
Email: gmdomke@fs.fed.us

File list

dcwd_vol_C_uncertainty.csv

Description

dcwd_vol_C_uncertainty.csv – Data file containing the contribution of each source of uncertainty to downed coarse woody debris volume and carbon storage estimate and all sources of uncertainty combined. These are the data presented in Fig. 8 of the manuscript and include the type of uncertainty evaluated (i.e. volume or carbon; uncertainty_type), source of uncertainty (uncertainty_source), plot number (plot), median (median), 25% confidence level (CI_25), 75% confidence level (CI_25), 2.5% confidence level (CI_2.5), 97.5% confidence level (CI_97.5). Units for volume are m^3 ha^{-1} and units for carbon are Mg C ha^{-1}. Additional information about the plots and how the data were collected are provided in O'Connell et al. (2015) and USDA Forest Service (2017).

Literature Cited

O'Connell, Barbara M.; LaPoint, Elizabeth B.; Turner, Jeffery A.; Ridley, Ted; Pugh, Scott A.; Wilson, Andrea M.; Waddell, Karen L.; Conkling, Barbara L. 2015. The Forest Inventory and Analysis Database: Database description and user guide version 6.0.2 for Phase 2. U.S. Department of Agriculture, Forest Service. 748 p. [Online]. Available at web address: http://www.fia.fs.fed.us/library/database-documentation/.

USDA Forest Service. 2017. Forest Inventory and Analysis national core field guide. Volume 1: Field data collection procedures for Phase 2 plots. Version 7.2. Washington, D.C.: U.S. Department of Agriculture, Forest Service. 433 p. https://www.fia.fs.fed.us/library/field-guides-methods-proc/.
